# Infection Prevention and Control in Asia: Current Evidence and Future Milestones

**DOI:** 10.1093/cid/cix071

**Published:** 2017-04-29

**Authors:** Anucha Apisarnthanarak, Linda M Mundy, Terapong Tantawichien, Amorn Leelarasamee

**Affiliations:** 1 Division of Infectious Diseases, Thammasat University Hospital, Pratum Thani, Thailand;; 2 Luitpold Pharmaceuticals, Inc, Norristown, Pennsylvania; and; 3 Division of Infectious Diseases, Chulalongkorn University Hospital, and; 4 School of Medicine, Siam University, Bangkok, Thailand

**Keywords:** nfection prevention, Asia, Asia Pacific, resource-limited setting, healthcare associated infections

The Asia-Pacific region is a geographic source for emerging infectious diseases, including multidrug-resistant (MDR) organisms (MDROs) and pathogens with pandemic potential. Risks for emerging infectious diseases in this geographic region are complex and are presumed to include ecological, socioeconomic, and technological processes favorable to microbial transmission dynamics. In resource-limited settings, relative to resource-adequate settings, there continues to be a paucity of data in support of infection prevention and control, and patient safety interventions to ensure that regional, if not national, healthcare systems work effectively to improve infection prevention and control interventions. In addition, several viral pandemics and annual influenza strains have originated in the Asia-Pacific region, which, together, has global implications for population health.

The prevention, control, and reporting of MDROs and healthcare-associated infections (HAIs) benefit from effective data dissemination plans and harmonized surveillance systems. HAIs compromise patient safety because they are preventable and serve as a reservoir for MDROs, which are associated with excess length of hospital stay, increased antimicrobial drug exposure, and excess costs [1–5]. In the Asia-Pacific region, the risks of HAIs have been estimated to be 2–20 times higher than in developed countries, with up to 25% of hospitalized patients reported to have acquired infections [4]. Although infection prevention and control is well recognized in the Asia-Pacific region, there are inconsistencies in the quality of evidence and dedicated resources to enhance current infection prevention practices, surveillance, and patient safety [4–6]. Existing evidence gaps include education, organizational and cultural barriers, physician and nursing champions, administrative support, infrastructure, fiscal resources, and key leadership that endorse implementation of infection prevention and control as a core component of patient safety programs across the Asia-Pacific region [6, 7].

This issue of *Clinical Infectious Diseases* focuses on 3 key themes of infection prevention and control in healthcare settings across the Asia-Pacific regions: (1) epidemiology and evidence to support prevention and control interventions, (2) enhancements to infection prevention and control in healthcare settings, and (3) practices associated with the containment of emerging infectious diseases and outbreaks. The epidemiological data and evidence to support prevention and control interventions include 2 national survey studies for best practices and 4 epidemiology studies. A survey from Thailand and Japan relative to the United States compared evidence-based practices for prevention of HAIs and identified a modifiable gap associated with quality improvement for hospitals in these Asian-Pacific countries.

A second national survey focused on policy, process, and outcomes associated with methicillin-resistant *Staphylococcus aureus* (MRSA) and MDR *Acinetobacter baumannii* interventions in Thailand. Higher compliance (>75%) with bundled approaches was associated with reduction in MRSA, where containment of MDR *A. baumannii* necessitated bundle compliance along with additional infection control interventions. Four other publications describe the complex clinical factors, molecular epidemiology, and transmission dynamic associated with prevalent and emerging MDROs in Singapore and South Korea (eg, MRSA, community-associated MRSA, and carbapenem-resistant Enterobacteriaceae). Together, these studies provide information on the epidemiology of infection prevention and control and the transmission dynamic of MDROs in Asia-Pacific settings.

The second theme in this issue on infection prevention and control in the Asia-Pacific region is the innovative use of molecular diagnostics, electronically linked administrative data, and information technology to enhance infection prevention and control. Findings from 4 meta-analyses or network meta-analyses demonstrate the reduction in central catheter–associated bloodstream infection with the use of minocycline-rifampin–impregnated central venous catheters; the effects of antibiotic stewardship on outcomes including antibiotic consumptions, mortality rates, and the reduction in MDROs; infection control–specific interventions associated with control of specific MDR gram-negative pathogens in intensive care units; and the potential role of the enteric microbiome in postoperative complications.

Together, these studies also provide strong methodological approaches for the use of network meta-analyses, which permitted assessment of these exposure-response relationships inclusive of potential confounding factors. In addition, a report from Singapore describes the use of an electronic medical record–linked administrative database that included language selectively of specific terms, using text parsing of chest radiographic reports to identify evidence of pneumonia. Use of this electronic medical record–linked data source in a case validation of hospitalized adults with community-acquired pneumonia provides an enhanced method for conducting real-world evidence studies that can include enhanced case detection, diagnostic evaluations, and pathogen-specific treatment patterns in future studies. Finally, the real-life feasibility of using the Xpert MTB/RIF assay (Cepheid) in clinical practice in a tuberculosis-prevalent country was reported from Thailand. Results suggest the potential role of this molecular diagnostic method in the containment of MDR tuberculosis in the Asia-Pacific region.

The third theme is practices associated with the containment of emerging infectious diseases and outbreaks in Asia-Pacific countries. Two publications describe successful containment for emerging infectious diseases, inclusive of lessons learned from the 15-year Hong Kong infectious diseases preparedness experience and the experience of zero secondary chain transmission of Middle East respiratory syndrome coronavirus in Thailand. Together, these 2 studies emphasize the key message that control of emerging infectious diseases is feasible and sustainable in these geographic regions.

Finally, 2 interesting outbreaks are reported in this issue. The first is a large food-borne outbreak of *Streptococcus agalactiae* infection in Singapore. Findings imply a potential role of regional collaboration to control *S. agalactiae,* because molecular epidemiological data were linked to the zoonotic spread from farmed freshwater fish. These fish share phylogenomic analysis of ST238, similar to human and fish isolates from Thailand and Hong Kong. A second report confirms a pseudo-outbreak of *Bacillus* spp. bacteremia linked to contaminated hospital linen, with emphasis on the benefit of good hygienic practice in central supply and sterilization services. These collective efforts complement existing evidence for the advancement of infection prevention science in the Asia-Pacific region.

Healthcare in the Asia-Pacific region has had notable milestones in infection and prevention control. The publications in this issue support future work focused on continued generation of evidence and dissemination of data, ongoing microbial surveillance, and implementation, if not adoption, of effective prevention strategies to contain the spread of MDROs and key emerging infectious diseases in the region. We thank the Infectious Diseases Association of Thailand for its partial sponsorship of this issue and share our hope that the collective experience from the study contributors reporting work in this issue will serve as a 2017 platform for improvement in the science and practice of infection prevention in the Asia-Pacific region and in other resource-limited settings.

## Notes

***Supplement sponsorship*.** This article appears as part of the supplement “Infection Prevention in Asia Pacific,” sponsored by the Infectious Diseases Association of Thailand (IDAT) with additional author sponsorship.

***Potential conflicts of interest.*** All authors: No potential conflicts. All authors have submitted the ICMJE Form for Disclosure of Potential Conflicts of Interest. Conflicts that the editors consider relevant to the content of the manuscript have been disclosed.
